# Comparative health systems research in a context of HIV/AIDS: lessons from a multi-country study in South Africa, Tanzania and Zambia

**DOI:** 10.1186/1478-4505-5-13

**Published:** 2007-10-30

**Authors:** Suraya Dawad, Nina Veenstra

**Affiliations:** 1 Health Economics and HIV/AIDS Research Division, University of KwaZulu-Natal, Durban, South Africa

## Abstract

Comparative, multi-country research has been underutilised as a means to inform health system development. South-south collaboration has been particularly poor, even though there have been clearly identified benefits of such endeavours. This commentary argues that in a context of HIV/AIDS, the need for regional learning has become even greater. This is because of the regional nature of the problem and the unique challenges that it creates for health systems. We draw on the experience of doing comparative research in South Africa, Tanzania and Zambia, to demonstrate that it can be useful for determining preconditions for the success of health care reforms, for affirming common issues faced by countries in the region, and for developing research capacity. Furthermore, these benefits can be derived by all countries participating in such research, irrespective of differences in capacity or socio-economic development.

## Introduction

HIV/AIDS is having a major impact on health systems in sub-Saharan Africa (sSA), most of which have in the last three decades also undergone a string of health care reforms aimed at improving efficiency, effectiveness and equity. Reforms have meant substantial change in both what is done and how it is done, in other words, change in both policies and institutions [1]. They have been complicated by the additional layer of complexity which HIV/AIDS has brought. This dual challenge of health care reform and HIV/AIDS faced by countries in the region suggests scope for joint learning. More generally speaking, there have also been calls for more comparative, multi-country research that will contribute to health system development [see for example [2]]. A recent review of health systems research publications demonstrated that only 10% of such papers make reference to multiple countries [3]. The review concluded that south-south collaboration in health systems analysis is particularly poor.

This commentary advocates for comparative, multi-country research to assist countries in sSA in developing their health systems in a context of HIV/AIDS. It does this in two ways. Firstly, it examines the use of comparative, multi-country health systems research. It considers the insights we might obtain from such research and, in particular, the rationale for joint learning to manage the impacts of HIV/AIDS. Secondly, this paper elaborates on the lessons learned from our experience of conducting comparative research in South Africa, Tanzania and Zambia. This research was designed to elicit challenges in the functioning and development of health systems in relation to HIV/AIDS. Although only a pilot study, the research revealed some unexpected benefits through its focus on these three countries.

## Why comparative research?

'Comparative health systems research' could be defined a number of ways and so it is necessary to clarify the definition employed for the purposes of our research and now for that of this commentary. Firstly, 'comparative' implies a comparison. Interestingly, past research indicates comparisons have not always been across countries – in some instances policy and programme analysis has looked at whether lessons are transferable from the education sector to the health sector, for example [4]. While such approaches are not without merit, this commentary focuses on more commonly encountered endeavours to develop shared learning across countries in a particular region. Secondly, by 'health systems research' we are looking specifically at research on the functioning of health management structures and processes which have been the subject of health system reforms. This is in contrast to research which concentrates on health facilities and their role in service delivery.

Using this definition, there is some experience to go by, since multi-country, comparative health systems research has been used to investigate a diverse range of health system issues in sSA including decentralisation, community financing and regulation of private sector activity, to name just a few [see [5-7]]. Other studies have looked at a broader range of health system characteristics in various contexts to see how these influence clinical outcomes [see for example [8] in relation to maternal health outcomes]. The question that we have to answer, before turning more specifically to the challenge of HIV/AIDS, concerns the contribution of comparative research to our insights on health system functioning. In other words, what is the rationale for doing health systems research across more than one country, given the potentially difficult logistics and much greater expense?

In most cases, the rationale for choosing to study a number of countries in the region is that the variation in context allows one to better understand the necessary preconditions for the success of national policies. In the case of community financing, for example, contextual factors, the design of different schemes, processes used in implementation, or the mix of actors involved, may all explain the pattern of equity impacts observed [6]. Other studies have pointed out how the aims and types of health care reforms in sSA have often been similar, but that these are implemented in different contexts and using different approaches, providing unique opportunities to understand factors influencing the success of reforms [9]. Such reasoning concurs with a 'realist' approach to evaluation which replaces the question of 'what works?' with the question of 'what is it about this programme or policy that works for whom in what circumstances?' [10]. This approach therefore focuses on the mechanisms through which a programme, policy, or reform strategy might work in relation to associated contexts.

While the 'realist' approach to evaluation constitutes the most common rationale for doing comparative research, there are other important benefits that can be derived. In particular, comparative research has been identified as a method to build cross-country research capacity [3]. Examples have been documented of research networks whose priority it has been to strengthen national research by combining and sharing knowledge and experience [11]. This might seem like a less important objective of comparative research, but without adequate research capacity, policy makers will not have access to sound information on which to base decisions and the potential for shared learning will be lost. Researchers stand to benefit from such research by developing their skills around elements such as research design, implementation and analysis. For this to occur, however, research has to be conducted by researchers within their respective countries [3].

Given these benefits of comparative research, we argue that this approach is ideally suited to examining the interface between HIV/AIDS and health system functioning. This is because HIV/AIDS is arguably the most challenging contextual factor that health systems are currently facing. The epidemic increases the demand for health care, while impeding household's ability to access care and worsening the human resource crisis. These stresses may be felt higher up in the system through various means including the establishment of new structures, the centralisation of management, more vertically structured programmes and an increase in donor activity. We hypothesize that how a health system copes (or indeed doesn't cope) with such stresses depends on a number of health system factors operating in tandem with other contextual factors. For example, the structure of the health system and the nature and extent of health reforms undertaken may all affect the ability of the health system to respond appropriately. Socio-economic factors, on the other hand, can impose additional restrictions on health systems, not least of all through limiting the amount of resources allocated to them.

Additional motivation is provided for conducting comparative research if we consider how little experience we have in dealing with HIV/AIDS. The pandemic is a threat to health systems development unlike any other previously experienced, partly because of its long wave nature and widespread impacts. Capacity building is urgently needed to facilitate management strategies that are responsive to challenges posed by the pandemic, and comparative research can provide such opportunities. Researchers need to develop the means to obtain timely information on health system impacts. Policy makers then have to become skilled at translating this information into appropriate action, to avoid forfeiting any progress made in developing and reforming the health system. The relationship between researchers and policy makers is clearly also fundamental in this regard.

Unfortunately, making a clear case for comparative research for health systems analysis in a context of HIV/AIDS does not detract from the difficulties associated with actually carrying out such research. The irony is that comparative research is most useful where many variables interact, yet it is precisely under these circumstances that methodological challenges abound [11]. The additional layer of complexity that HIV/AIDS has brought to health system development in sSA brings with it the need for developing analytic frameworks that yield data that are comparable across countries.

## Health systems in a context of HIV/AIDS: lessons from South Africa, Tanzania and Zambia

In view of the potential insights to be obtained from comparative research in a context of HIV/AIDS, the Health Economics and HIV/AIDS Research Division (HEARD) at the University of KwaZulu-Natal in South Africa decided in 2004 to explore a range of health sector issues using such research and through partnerships with other research organisations in the region. The resulting research project has been a collaboration between HEARD, the Muhimbili College of Health Sciences in Tanzania and the Department of Economics at the University of Zambia. While HEARD played a key role in initiating and co-ordinating the research, the design, implementation and analysis of the research itself was an entirely collective effort, with each partner responsible for obtaining insights from their own county. The research commenced with a one-year pilot study in 2005, the objectives of which were to:

1. Better understand how health systems in South Africa, Tanzania and Zambia are functioning in the context of HIV/AIDS ie. looking at HIV/AIDS as a contextual factor in health system development and reform

2. Establish partnerships between collaborating research institutions and build the capacity of researchers to do regional research

3. Establish and strengthen relationships with government departments

This pilot has subsequently and intentionally informed a longer term research agenda and this commentary captures some of our reflections and learning from this process.

Insights from the research are written from the South African perspective, to highlight the benefits that a middle income country can derive from doing comparative research with less well off countries in the same region. However, these benefits were not defined by South African researchers alone. Certain concerns became apparent during interactions with study participants in South Africa – managers at various levels of the health system. These were later raised as issues of interest at 'researcher meetings', essentially forums for sharing results and experiences that were attended by all collaborating partners. It was at these meetings and during the course of sharing results that the true benefits of the research became apparent.

As comparative research seeks to explain how contextual factors shape health system responses and why, a key consideration for designing such research will always revolve around the choice of countries to compare. Chosen countries should ideally have some contextual factors that are similar and some that are different. In our research some of these factors were mapped out during the planning phase – Table 1 documents the similarities and differences that contributed to our decision to conduct research in South Africa, Zambia and Tanzania. Other reasons for our choice of countries stemmed from logistical concerns rather than the mapping of contextual factors. Most importantly, interested research partners had to be identified and this was done through established links in the region. Finally, not all contextual factors were considered in planning and so the importance of some only became apparent later on in the course of the research. For example, the different timeframes for health sector reform across the three counties was quite different, since South Africa was only able to commence reforms after the demise of apartheid in 1994. This factor had a marked influence on the ability of different countries to accommodate the impact of HIV/AIDS on health sector management structures and their functioning.

**Table 1:** Contextual factors contributing to the choice of countries

**Similarities across chosen countries**	**Differences across chosen countries**
• Within the region of sub-Saharan Africa • High HIV prevalence – between 6.5% in Tanzania and 18.8% in South Africa [12] • Reforms aspiring to model of decentralised management and primary health care	• Socio-economic status and health sector capacity – amount spent on health ranging between US$29 PPP (Tanzania) and US$669 PPP (South Africa) per capita [13] • Extent of health sector reforms – South Africa and Zambia described as 'radical reformers' because of the comprehensive reform strategies adopted in the health sector [14]

A second consideration concerns the use of comparative frameworks and data collection instruments. Such frameworks enable a more nuanced understanding of the relevance of experiences across countries [15]. More advanced instruments have incorporated multiple health system dimensions to enable comparisons among a range of countries, from industrialised to very poor, and from entrepreneurial to socialist. The selection of 3 countries for our research did not necessitate such broad health system comparison. In our case it was a matter of defining several key areas of health care reform that could have interfaced with HIV/AIDS stresses in the health sector, and of devising tools that could collect comparable data. In the pilot phase of our research project the comparison focussed on three key theme areas: health system structures, decentralisation processes, and health sector partnerships. These essentially covered a limited set of structural and financing policies and reforms.

The choice of countries for our research yielded interesting insights and a significant degree of two-way learning. This learning challenged a common assumption that countries with a higher level of socio-economic development would automatically be in a better position to cope with the challenges posed by HIV/AIDS, and in so doing provide lessons for less developed countries. We found in many cases the opposite applied. For example, Tanzania and Zambia are both countries that have historically received large amounts of donor aid. As a result, and not surprisingly, they have both had significant experience with different ways of managing partners in the health sector and with measures to coordinate external resources constituting a fundamental component of their health sector reform [see for example [16]]. The means to ensure that partners' support is positive, although not always functional, was nonetheless in place before the influx of support for HIV/AIDS activities. The health system structure also facilitated a more coordinated approach to managing health services, with some districts small enough for 'everyone to know everyone'.

South Africa, on the other hand, has only experienced a massive influx of support with the advent of HIV/AIDS in the last decade. The South African health system has not been prepared for this, and so NGO and donor co-ordination was found to be somewhat chaotic. In some instances it resulted in conflict between different levels of government, with the clearest illustration of this being when monies were granted to one province by the Global Fund without the consent of the National Department of Health [17]. The policy documents suggest that donor coordination should happen at the national level, where in practice managers felt that this was inappropriate and that people more in tune with on the ground activities should have more control. As health districts in South Africa are larger than in other countries, managers often didn't know which NGOs were operating in their districts, so creating an even greater need for formal coordination mechanisms. Hence the South African health system was still learning how best to manage the external support that was part of the context created by HIV/AIDS.

HIV/AIDS has also interfaced with other contextual factors, such as the political context, to impede the development of health systems. In South Africa, health care reforms and decentralisation in particular, could only truly be implemented post 1994. This process was understandably slow, with legislative effect given to transformation within the health sector by the National Health of 2003, most sections of which came into effect on 2 May 2005 [18,19]. This Act lays out the functions of national and provincial health departments, health districts, and structures supporting these, such as health councils and consultative forums. Prior to the Act, significant effort went into establishing relevant, but sometimes temporary, structures to manage district health services. This was also, however, the time that HIV/AIDS impacts were starting to be felt in the sector. Hence tension developed between the immediate need for the health system to deliver through the existing centralisation model and the longer term developmental objective of a strengthened local government through decentralisation [see also [20]]. At the time of our research, district health managers were struggling with the task of defining district health needs, but were also not given the autonomy to place HIV/AIDS in the context of other health priorities.

The Tanzanian and Zambian health systems, on the other hand, have had a much longer history of implementing decentralisation, hence placing them in a more advantageous position to cope with the pressures of HIV/AIDS. In both these countries, reforms have progressed to the point where communities are involved in identifying their needs. Health priorities emanate from the village level, and are fed up to the ward level before being compiled at the district level. While our research indicated a certain degree of mediation from above during this process, the system was essentially well established. It allowed communities to have a much stronger role in the health system, even in the presence of HIV/AIDS. This involvement is said to be essential for the development of 'innovatory culturally acceptable solutions' to health problems and is constantly aspired to as a fundamental element of the primary health care approach [21-23].

While differing experiences across countries have provided many opportunities for learning, the identification of similar issues or concerns has provided health system managers with reassurance that they are not alone in their endeavours to cope with the changes resulting from HIV/AIDS. This benefit should not be underestimated in contexts where the challenges faced by these managers have the potential to become entirely overwhelming. Unless these managers feel supported and realise that there are things they can do to respond to the HIV/AIDS stresses imposed on the health system, they are at risk of feeling incapable of doing anything constructive. They may even deny the problems, if the information they are presented with is too threatening [24].

In South Africa, HIV/AIDS has fuelled long-standing mistrust between policy makers and researchers/civil society. This is evidenced, for example, by the positioning of the Treatment Action Campaign (TAC) as the 'enemy', when in fact its purpose was always to support government [25,26]. Our research helped to alleviate some of the mistrust between researchers and government officials in South Africa and to stimulate a sense of purpose by providing managers with reassurance that the issues raised are not a reflection of their poor performance, but are similar to those faced by other countries in the region. Furthermore, the research highlighted that there are lessons to be learnt in responding to the changing context. Initially many policy makers were reluctant to meet with us and some were quite defensive in their responses to our questioning. However, when we presented our results back to them, they became far more interested and involved in the debates. They wanted to know how health systems in Tanzania and Zambia were coping with HIV/AIDS stresses and were very supportive of us proceeding with the next phase of the research. As such, in South Africa we felt that we achieved our objective of improving relationships with government departments, one which was not as relevant in the other countries participating in the research.

One further benefit of comparative research in a context of HIV/AIDS is south-south capacity development. All too often there is a belief that the north holds the knowledge and capacity that should inform the south; this has historically resulted in much north-south collaboration [3]. However, we argue that countries in sSA are experiencing a range of common challenges for which they have to develop their own solutions, calling for a greater degree of collaboration across the region. HIV/AIDS is clearly one of these challenges because of the differing extents and features that the epidemic has assumed in different regions of the world. One of the objectives of our research was to build the capacity of researchers to do regional research and there were two elements of our approach that were useful in this regard. Firstly, country teams were responsible for research within their own countries, where their knowledge of the context could be utilised to its fullest potential. Secondly, country teams contributed a diverse range of expertise (in economics, public administration, public health, population studies and anthropology) to research planning and analysis, resulting in a truly multi-disciplinary approach to understanding the issues.

## Conclusion

Comparative, multi-country health systems research, particularly that involving south-south collaboration, has previously yielded a number of benefits which were confirmed through our experience of conducting such research in South Africa, Tanzania and Zambia. In particular, comparative research was found to be useful for determining preconditions for the success of health care reforms, affirming issues that are generalisable to countries in the region, and developing research capacity. More importantly, these benefits were enjoyed by all countries participating in the research, irrespective of differences in capacity or socio-economic development. However, comparative research has thus far been fairly limited in scope.

The devastating scope of the HIV/AIDS epidemic in sSA requires greater efforts to conduct comparative health systems research across countries. The regional nature of the challenge and the unique features of the epidemic call for new approaches to learning. Researchers in the region need to work together to develop and share the capacity required to understand how HIV/AIDS changes the context in which health systems are having to develop. This is one crucial step on the path toward achieving our vision for health systems in Africa.

## Competing interests

The author(s) declare that they have no competing interests.

## Authors' contributions

Both authors participated in the conception, general idea and writing up of the commentary. All authors read and approved the final manuscript.
